# The role of biological nitrogen fixation in polyhydroxybutyrate production from methane by methane-oxidizing bacteria: a review of metabolic routes and yield enhancement

**DOI:** 10.3389/fmicb.2025.1614676

**Published:** 2025-11-14

**Authors:** Jin Hui Xie, Jia Ying Xin, Li Rui Sun, Tian Yu Cui, Hai Xin Bi, Yan Wang, Jian Xiong Zhang

**Affiliations:** 1 Key Laboratory of Food Science and Engineering, Harbin University of Commerce, Harbin, China; 2 State Key Laboratory of Carbonyl Synthesis and Selective Oxidation, Lanzhou Institute of Chemical Physics, Chinese Academy of Sciences, Lanzhou, China

**Keywords:** methane-oxidizing bacteria, poly-β-hydroxybutyrate, biological nitrogen fixation, nitrogen gas, metabolic engineering, bioprocess optimization

## Abstract

In recent years, the synthesis of Polyhydroxybutyrate (PHB) using methane-oxidizing bacteria (MOB) to convert one carbon and N_2_ resources has received much attention. In addition, nitrogen fixation by nitrogen gas (N_2_) has an effect on the metabolic pathways of MOB. Although progress has been made in the basic metabolic pathways and the role of key enzymes, there are still many challenges, such as explorating the synergistic mechanism of one carbon and nitrogen and how to optimize the cultivation conditions to increase yield and reduce costs. This paper is concerned with the biological characteristics of methanogens and their role in the metabolism of one carbon and N_2_ resources. In addition, it introduces the optimization of their PHB synthesis capacity by new technologies in the field of metabolic engineering. The aim of the paper is to provide a theoretical basis for solving plastic pollution and realizing renewable utilization of resources.

## 1 Introduction

Amid growing concerns over climate change and plastic pollution, the development of sustainable alternatives to conventional plastics has become imperative. PHB is a polyester that exhibits favorable biocompatibility and biodegradability. In recent years, MOB applications ranging from environmental remediation to bioplastic synthesis are a hot topic (Puyol et al., 2017). PHB synthesis of MOB based on one carbon and N_2_ resources due to the efficient use of simple carbon and N_2_ sources, not only does it advance the knowledge of microbial metabolic engineering, but it also highlights the importance of reusing resources and proserving new ideas for producing bioplastics.

MOB exhibits a distinctive metabolic capacity to proliferate and metabolize methane (Evans et al., 2019), a prevalent one carbon resource, as a substrate. Under specific circumstances, intracellular metabolic pathways can be redirected toward PHB synthesis (Lopar et al., 2013). In recent years, significant progress has been made in elucidating the fundamental metabolic pathway for PHB synthesis by methanotrophic bacteria utilizing one carbon resource. However, the optimal methodology for enhancing this process remains an area that requires further investigation. N is an essential element for microbial growth, and it plays an important role in the metabolism of MOB and the synthesis of PHB. The growth rate, enzyme activity and carbon metabolism partitioning of MOB may be influenced by the supply of N_2_ in different forms and concentrations (Shukla et al., 2013). The precise regulatory mechanism of nitrogen resources in the synthesis of PHB in MOB remains unclear, and further investigation is warranted, particularly with regard to the potential synergistic effects with one carbon resources. Salem (Salem et al., 2021) demonstrated that MOB using methane and N_2_ as substrates were able to synthesize PHB at a relatively low cost and with minimal impact on the environment. Consequently, the study of the metabolic processes of MOB under one carbon and N_2_ resource conditions is a significant objective in the domain of bioengineering, with the aim of enhancing the efficiency of PHB synthesis.

This paper presents a new perspective on a carbon and N_2_ resource and also explores the process of PHB synthesis by different types of MOB, the selection of carbon and nitrogen sources, and the latest technologies in the field. We specifically address the biochemical pathways governing PHB synthesis from methane, methanol, and CO_2_; evaluate the role and impact of N_2_ fixation on PHB yield; discuss critical factors affecting synthesis efficiency; and analyze advanced strategies in genetic engineering and bioreactor design for process enhancement. By integrating recent advancements and identifying persistent research gaps, this work aims to establish a clearer theoretical basis for leveraging MOB in sustainable bioplastic production, contributing to both academic knowledge and industrial applications in renewable resource utilization.

## 2 A comprehensive overview of MOB

### 2.1 The classification and characterization of MOB

MOB are a group of microorganisms that specialize in methane oxidation metabolism. Aerobic MOB are commonly found in a variety of environments, including soils (Shen et al., 2015), wetlands and aquatic systems. In oxygen-rich environments, these bacteria are able to oxidize methane to carbon dioxide. These microorganisms play a role in carbon fixation and carbon cycling, contributing to improving environmental quality (Llamas et al., 2023), controlling greenhouse gas concentrations and promoting plant growth. Anthropogenic methane emissions are usually oxidized by anaerobic MOB in anoxic environments. These bacteria utilize methane as an electron donor through a symbiotic relationship with sulfate-reducing bacteria, which further convert methane into carbon dioxide and cellular biomass. Therefore, studying the taxonomy of MOB is crucial for understanding global climate change and developing effective environmental strategies (Wei et al., 2022).

According to whether the oxygen in the environment is used as an electron acceptor, it can be divided into two categories: aerobic methane oxidizing bacteria and anaerobic methane oxidizing bacteria (Table 1). At present, aerobic methanotrophs are mainly divided into type I, type II, and type X according to the carbon source assimilation path, cytoplasmic membrane structure, fatty acid carbon chain length and dormancy period of the strains.

**Table 1:** Classification of methane-oxidizing bacteria and archaea.

**Metabolic types**	**Type**	**Phylum (Class)**	**Genus (Species)**
Aerobic methane-oxidizing bacteria	Type I, X	γ-Proteobacteria	*Methylomonas, Methylobacter, Methylosarcina, Methylomicrobium, Methyllohalobius, Methylosphaera, Methylosoma, Methylothermus, Methylocaldum, Methylococcus*
	Type II	α-Proteobacteria	*Methylosinus, Methylocystis, Methylocella, Methylocapsa, Methyloferula*
	Others	Verrucomicrobia	*Methylokorus infernorum, Acidimethylosilex, Fumarolicum, Methylocacida kamchakensis*
Anaerobicmethane-oxidizing bacteria	Archaea	ANME	ANME-1, ANME-3, ANME-2, ANME-2b, ANME-2c
			ANME-2d
		Marine benthic group D	Unclassified
	Bacteria	*Phylum NC10*	*Candidatus Methylomirabilis oxyfera*

Type I MOB belong to γ subclass of *Proteobacteria*, which are characterized by: (1) the cytoplasmic inner membrane penetrates the cells to form multi-bubble disc-like bundles; (2) The carbon assimilation pathway is RuMP pathway; (3) The carbon chain lengths of characteristic fatty acids are 14C and 16C. Type II MOB belong to α-subclass of *Proteobacteria*, which are characterized by: (1) the cytoplasmic inner membrane is aligned in a linear arrangement on the periphery of the cell; (2) The carbon assimilation pathway is serine pathway; (3) The carbon chain length of characteristic fatty acids is 18C. X-type MOB include *Methylococcus* and *Methylocaldum*. X-type MOB have the characteristics of both type I and type II MOB. The carbon assimilation pathway is mainly RuMP pathway and serine pathway. X-type bacteria have strong heat resistance.

### 2.2 Ecological roles

MOB use methane as one carbon source and utilize aerobic and anaerobic metabolic pathways to convert the greenhouse gas methane (Sun et al., 2018) into carbon dioxide and water, thereby reducing atmospheric methane concentrations. This process can be achieved by rational management of soil microbial communities, thereby reducing methane emissions during agricultural production (Samanta and Sani, 2023).

## 3 Biochemical pathways and influencing factors of PHB synthesis

### 3.1 PHB synthesis in MOB metabolizing one-carbon sources

#### 3.1.1 PHB synthesis from methane

The intermediate metabolites produced during the MOB conversion process can be channeled into PHB biosynthesis through specific enzymatic processes. However, the efficiency of PHB synthesis is strongly dependent on metabolic regulation and key environmental conditions. These influential factors include temperature, pH, and the availability of essential nutrients throughout the metabolism (Shimizu, 2016). Therefore, a thorough exploration of the synthesis mechanism is critically important. Such investigation not only deepens our understanding of the scientific underpinnings of this bioconversion but also establishes a theoretical basis for practical implementation. This advancement is crucial for promoting the development and utilization of sustainable bio-based materials (Reichert et al., 2020).

#### 3.1.2 PHB synthesis from methanol

Methanol, a simple alcohol, has emerged as a significant substrate for PHB production in methanotrophic bacteria, primarily due to its status as a readily available and efficiently converted carbon source (Das et al., 2021). Within the metabolic network of these bacteria, methanol fulfills a dual role, acting as both an energy source and a direct precursor for PHB biosynthesis. The conversion pathway initiates with the oxidation of methanol to formaldehyde, a reaction catalyzed by the enzyme methanol dehydrogenase. Formaldehyde is subsequently converted to formic acid (Dorokhov et al., 2015), which is then oxidized to carbon dioxide by formate dehydrogenase, liberating energy in the process. The metabolic intermediates from these reactions are ultimately directed into the PHB synthesis pathway. This biosynthesis involves three key enzymatic stages: ketoacyl synthesis, polymerization, and deacetylation (Sagong et al., 2018). Specifically, methanol-derived metabolites are channeled to form ketoacids, which are then converted into β-ketoacids by β-ketoacid synthase before undergoing polymerization into the final PHB polymer. Consequently, the overall efficiency of this synthesis is considerably influenced by environmental conditions, including nutrient salt concentration, pH, and temperature.

The metabolic pathway for synthesizing PHB from methanol via MOB represents a sustainable alternative to conventional polymer production. This bioconversion offers a promising biotechnological strategy for mitigating environmental challenges. As research in this field advances, future efforts to optimize the coupled process of methanol assimilation and PHB biosynthesis are critical. Such optimization is anticipated to facilitate the large-scale implementation of this technology, thereby contributing significantly to the achievement of sustainable development goals.

#### 3.1.3 Synthesis of PHB from carbon dioxide

The biosynthesis of PHB from methane by MOB involves a multi-step biochemical pathway, primarily comprising methane oxidation, central metabolite formation, and polymer synthesis (Hwang et al., 2020). The process is initiated by MMO, which catalyzes the oxidation of methane to methanol. Methanol is subsequently dehydrogenated to formaldehyde, which serves as a key branch point in the metabolism. A portion of formaldehyde is assimilated into central carbon metabolism via pathways like the ribulose monophosphate or serine cycle, generating metabolic precursors. These precursor molecules are then channeled into the PHB synthesis pathway. This involves a series of enzymatic reactions, including condensation by ketoacyl-thiolase, reduction by acetoacetyl-CoA reductase, and finally polymerization by PHB synthase. The entire process is driven by energy and reducing equivalents generated from the oxidation of another portion of the formaldehyde to CO_2_ (Zehnder and Brock, 1979). Ultimately, the PHB polymer accumulates within the bacterial cells as intracellular granules (Manoli et al., 2022).

### 3.2 Bio-utilization of one carbon and N_2_ resources

The biosynthesis of PHB in MOB proceeds through a defined metabolic pathway where methane is progressively converted into key intracellular intermediate metabolites, ultimately serving as precursors for PHB polymerization (Wang et al., 2020). A significant ecological advantage of certain MOB strains is their capacity for nitrogen fixation, allowing them to utilize N_2_ as a nitrogen source to sustain growth and metabolic activity (Nicholas, 1963). This capability is particularly beneficial for PHB production, as the synthesis process itself does not require large amounts of exogenous nitrogen. The ability to fix N_2_ can substantially reduce production costs and exemplifies the efficient cycling of matter and energy within these biological systems. A comprehensive investigation into the mechanisms of PHB synthesis from one carbon compounds and N_2_ is therefore of considerable importance. Such research not only deepens our understanding of the critical physiological role played by microorganisms in global biogeochemical cycles (Docherty and Gutknecht, 2012) but also establishes a vital theoretical foundation for developing efficient and environmentally benign biosynthetic technologies. This approach is anticipated to advance the sustainable development of the biomanufacturing industry, offering innovative solutions to pressing challenges such as resource scarcity and environmental pollution.

In addition to carbon sources, nitrogen sources have been shown to have a significant effect on PHB synthesis. The most commonly used nitrogen sources include ammonia, nitrate, urea, and N_2_ (Ismail, 2022). Several studies have demonstrated that the use of highly concentrated N_2_ sources and their optimal configuration can significantly increase the metabolic rate of microorganisms and accelerate the accumulation of PHB (Karasavvas and Chatzidoukas, 2020). These bacteria typically inhabit methane-rich environments and are able to efficiently utilize methane as the one carbon source, while showing excellent biosynthesis capacity under N_2_-limited conditions. In recent years, there has been a gradual increase in the number of studies on the synthesis of PHB by N_2_-sourced MOB. It was found that the growth rate, biomass concentration and PHB synthesis ability of mixed MOB could be significantly enhanced by implementing a cyclic culture model with nitrate and N_2_ (Salem et al., 2021). For instance, alternating nitrate and N_2_ cultures led to the stabilization of PHB synthesis capacity in the mixed colony after several cycles, with a maximum increase in PHB concentration of 0.64–1.27 times after nitrate incubation. Chau et al. (2022) elucidated the genomics and metabolic pathways of PHB synthesis in the MOB using molecular biology, metabolic engineering, and synthetic biology. For instance, several studies have demonstrated that the bacterial PHB synthesis pathway undergoes alterations in response to variations in N_2_ supply conditions, thereby effectively adapting the bacterial metabolic strategy to changing environments. Tays C achieved optimization of MOB in PHB synthesis by adjusting the conditions of carbon-to-nitrogen ratio, pH, and temperature, thereby increasing the overal productivity (Tays et al., 2018). The combination of N_2_ resources and MOB. The combination of N_2_ resources and MOB provides new perspectives and methods for PHB synthesis.

#### 3.2.1 Bio-use of one carbon resources

##### 3.2.1.1 Bio-use of methane

Following the initial absorption of methane into the cell by transporter proteins on the cell membrane, methane is oxidized by methane monooxygenase (MMO). The formation of methanol is the key reaction in the methane oxidation process and is directly related to the efficiency of subsequent reactions. The synthesized methanol will undergo further oxidation to formaldehyde and formic acid, and will eventually be converted to carbon dioxide (Qian et al., 2003). This process also requires the involvement of enzymes. The series of reactions enables the release of carbon from methane into CO_2_ while simultaneously providing energy for the growth of microorganisms. In conditions conducive to growth, such as those characterized by nutrient abundance or excess carbon sources, MOB is capable of converting a portion of the methanol into PHB. The synthesis of PHB is primarily accomplished through the synthesis and polymerization of short-chain acyl coenzyme A (Valentin and Steinbüchel, 1994). The synthesis of PHB is primarily achieved through the synthesis and polymerization of short-chain acyl coenzyme A. By utilizing the important carbon resource methane through a bio-based process, greenhouse gas emissions can be effectively reduced, the impact of climate change can be mitigated, and the resulting product can be reused to promote the carbon cycle (Yang et al., 2023).

##### 3.2.1.2 Bioavailability of methanol

The conversion of methanol by MOB is primarily reliant upon enzyme catalysis, which occurs in two steps. Initially, methanol is converted to formaldehyde by methanol dehydrogenase. Subsequently, formaldehyde is converted by formaldehyde dehydrogenase to formic acid, which eventually enters the methane synthesis pathway.

The conversion of methanol to other compounds is a complex process that requires a series of specific reactions and the involvement of certain enzymes (Shivhare et al., 2021). The initial step involves the reaction of methanol with inorganic phosphate under particular conditions, in the presence of enzymes, to produce methyl phosphate. This process requires the participation of key enzymes, such as β-hydroxy acid dehydrogenase and polymerase. Methyl phosphate then undergoes a series of enzymatic reactions, leading to the final conversion to β-hydroxybutyric acid. This final process requires enzyme catalysis to form the biodegradable polymer PHB and the polymerization of β-hydroxybutyric acid into PHB through esterification (Satti and Shah, 2020). MOB promotes the biodegradation of aromatic compounds during methanol utilization for waste purification (Czatzkowska et al., 2020). The metabolic efficiency of the synthesis of PHB from methanol by MOB is affected by a number of factors, including temperature, pH, oxygen concentration, and nutrient availability. Optimal temperature and pH conditions promote the growth of MOB and its metabolic activities, while also increasing the bioavailability of methanol. Oxygen is also important for methane oxidation and methanol production, while the proper configuration of nutrient salts can optimize the growth environment of microorganisms and enhance the synthesis of PHB.

##### 3.2.1.3 Bio-utilization of carbon dioxide

Within the metabolic network of MOB, carbon dioxide assumes a dual role: it is both a terminal metabolic by-product and a key precursor for biomaterial synthesis. The oxidation pathway begins with the conversion of methane to methanol, catalyzed by methane monooxygenase. Methanol is subsequently oxidized to carbon dioxide through a series of enzymatic reactions. Notably, MOB can reassimilate this CO_2_, along with other carbon sources like amino acids and fatty acids, for anabolic processes. This re-assimilation activates specific metabolic pathways that channel carbon toward the formation of biosynthetic precursors, ultimately leading to the synthesis of PHB within the cell.

As demonstrated by Sahoo et al. (2021) O_2_ to glycerol triphosphate via the Calvin cycle, with subsequent metabolic pathways yielding intermediates such as acetic acid and pyruvate. MOB is able to utilize these substrates to facilitate the conversion of CO_2_ to polymers. Synthesis of PHB is mainly dependent on β-hydroxybutyrate synthase. Under conditions where carbon is abundant but nitrogen is scarce, MOB synthesizes PHB through the process of enzymatically polymerizing precursors, this process serves a dual purpose, both as an intracellular energy reserve and a protective measure against external environmental stresses.

The transformation of CO_2_ into methane and the subsequent synthesis of PHB by MOB has been demonstrated to be an effective strategy for reducing atmospheric methane and CO_2_ concentrations, while concurrently converting CO_2_ into renewable biomaterials with substantial environmental sustainability (Muiruri et al., 2022). Notwithstanding the encouraging outcomes observed with MOB in its capacity to convert CO_2_ into PHB, numerous challenges persist in terms of achieving optimal yield and efficiency, thus hindering the fulfillment of the necessary standards for its application in industrial contexts (Samanta and Sani, 2023). Optimization of culture conditions and enhancement of strain resistance and stability are identified as pivotal strategies to foster the industrial implementation of MOB.

#### 3.2.2 Bio-utilization of N_2_

N_2_ is a ubiquitous element in nature, yet the majority of organisms do not utilize it directly as a source of nitrogen, it must be converted into a biologically usable form, a process called nitrogen fixation. Nitrogen is an essential component of proteins and nucleic acids. The availability of nitrogen has been demonstrated to directly affect the growth and metabolic activities of microorganisms. The bioavailability of nitrogen therefore often depends on environmental conditions or the metabolic capacity of a particular microorganism. This process relies on the action of nitrogenase that break the triple bonds in the nitrogen molecule to form ammonia, which provides the necessary source of nitrogen for cell growth (Koch, 2020). The status of nitrogen supply has been shown to affect the metabolic pathways and physiological state of the MOB. When nitrogen is in sufficient supply, metabolic activities in the cell are more active, which may promote the oxidation of methane and the production of related intermediates, thus providing more precursors for PHB synthesis.

#### 3.2.3 Status of biological nitrogen fixation technologies

The process of biological nitrogen fixation by certain microorganisms converts atmospheric N_2_ to ammonia, which is then used directly by plants as a source of nitrogen. Nitrogen-fixing microorganisms are usually classified as either rhizobia or cyanobacteria. However, recent studies have found that some MOB have nitrogen-fixing ability, such as *Methylococcus capsulatus* (Ahmadi and Lackner, 2024). This provides a new approach for MOB to synthesize PHB in nutrient-poor environments. The nitrogen-fixing capacity of MOB presents the potential for their growth and PHB synthesis under specific environmental conditions. Thevasundaram et al. (2022) demonstrated that MOB with nitrogen-fixing capabilities can obtain essential nitrogen through the nitrogen fixation pathway in nitrogen-deficient environments, thereby enhancing the efficiency of PHB synthesis. For instance, under conditions of high nitrogen limitation, specific MOB is capable of increasing the expression of nitrogen-fixing enzymes through metabolic adaptations to facilitate nitrogen fixation. Consequently, the issue of a nitrogen source required for normal growth was resolved, while the synthesis of PHB was simultaneously augmented. The utilization of MOB nitrogen fixation capabilities enables the bioconversion of methane and synthesis of PHB in nitrogen-source-poor environments, thereby reducing production costs. Furthermore, it offers the potential for developing biomanufacturing processes based on the co-utilization of methane and nitrogen, such as the construction of an efficient MOB-nitrogen-fixing bacterial co-culture system to achieve the synergistic utilization of one carbon and N_2_ resources and the production of high-value-added bioproducts.

From a practical standpoint, biological nitrogen fixation technology offers a more stable and cost-effective approach to PHB synthesis in MOB. In recent years, numerous studies have investigated methods to enhance the PHB synthesis and nitrogen fixation capacity of MOB, focusing on optimizing culture conditions and genetic engineering techniques. Rueda Hernández (2017) has demonstrated that the nitrogen fixation capacity of the bacterium can be enhanced by adjusting the medium composition, specifically by increasing the concentration of phosphate and trace elements, which in turn promotes PHB synthesis. Furthermore, there is a growing interest in modifying MOB to elevate the expression level of its nitrogen fixation genes through gene editing technology.

Research on the biological nitrogen fixation of MOB in PHB synthesis is still in its infancy; however, it has shown considerable potential for future applications. By enhancing the nitrogen fixation capacity of MOB, the growth environment can be optimized and the synthesis efficiency of PHB can be improved, while also further promoting the development of sustainable materials. As scientific and technological progress continues, it is anticipated that biological nitrogen fixation technology will assume an increasingly significant role in the synthesis of PHB from MOB (Kumar et al., 2021), thereby contributing to the promotion of environmental protection and sustainable development.

#### 3.2.4 Effect of nitrogen sources on PHB synthesis

While the empirical effects of nitrogen sources on PHB yield are well-documented, a deeper examination of the underlying regulatory mechanisms reveals a complex interplay between nitrogen assimilation, central carbon metabolism, and global cellular physiology. Moving beyond the general observation that nitrogen limitation enhances PHB accumulation, it is critical to analyze how different nitrogen sources exert distinct effects through specific metabolic and regulatory pathways.

The form of nitrogen provided fundamentally alters the biochemical cost of its assimilation. Ammonium (${\text{NH}}_{4}^{+}$) assimilation is the most energy-efficient, directly incorporating into amino acids via the glutamate dehydrogenase (GDH) or glutamine synthetase/glutamate synthase (GS/GOGAT) pathways. Nitrate (${\text{NO}}_{3}^{-}$) assimilation requires reduction to nitrite and then to ammonium before incorporation, consuming additional reducing equivalents (NAD(P)H and ATP). Biological nitrogen fixation is the most energetically costly process, consuming a substantial 16 ATP molecules per molecule of N_2_ fixed, alongside the obligatory production of H_2_. This high ATP demand directly competes with the energy requirements for PHB biosynthesis, providing a fundamental mechanistic explanation for the observed trade-off between growth rate and PHB yield when using N_2_. The cell must rebalance its energy budget, often at the expense of biomass proliferation.

N_2_ is a component of the atmosphere that constitutes approximately 78% of the air's volume. It is a cost-effective and readily available resource that can significantly reduce the cost of raw materials for PHB production and generate considerable economic benefits in large-scale industrial production (Du et al., 2012). The utilization of N_2_ as a source of nitrogen is consistent with the principles of sustainable development as it is a naturally occurring, renewable gas resource. Moreover, it curbs the overreliance on conventional chemical nitrogen sources, thus fostering a paradigm shift toward a more environmentally conscious and resource-efficient production model. N_2_ as a nitrogen source offers distinct environmental advantages over chemically synthesized alternatives. It circumvents the substantial energy consumption, wastewater generation, and potential ecological harm associated with industrial fertilizer production, thereby aligning with green production principles. Furthermore, the use of N_2_ can facilitate the enrichment of methane-oxidizing mixed consortia with high PHB-synthesis potential from complex environments like activated sludge (Jiang et al., 2016). These adapted microbial communities often exhibit broader substrate versatility, greater environmental resilience, and lower nutritional demands. These characteristics contribute significantly to reducing the overall cost of PHB production.

The use of N_2_ as a nitrogen source plays a critical role in the conversion of methane to PHB by MOB. An adequate supply of N_2_ enhances intracellular metabolic activity, promoting the oxidation of methane and increasing the production of key metabolic intermediates. MOB utilize methane as their primary carbon and energy source, and the presence of N_2_ within an optimized cultivation environment facilitates this conversion process, generating essential precursors for PHB biosynthesis (Wang et al., 2020). Specifically, metabolic intermediates such as organic acids provide the necessary carbon skeletons for the polymerization reaction that forms PHB (Mierziak et al., 2021). The presence of bioavailable nitrogen triggers specific genetic regulatory mechanisms within the cell (Velázquez-Sánchez et al., 2020), leading to the expression of enzymes that catalyze these biochemical reactions. This increases the overall rate and efficiency of the metabolic pathway leading to PHB accumulation. Furthermore, a stable intracellular environment is crucial for maintaining high enzymatic activity. Parameters such as consistent pH and ionic strength ensure that the enzymes involved in PHB synthesis function optimally, preventing the decline in activity that can result from environmental fluctuations.

Nevertheless, the application of N_2_ as a nitrogen source presents certain constraints. MOB cultures relying solely on N_2_ frequently demonstrate reduced growth rates and lower final biomass concentrations compared to those supplemented with fixed nitrogen sources. This limitation stems from the biological nitrogen fixation process, which is energetically costly for the cell as it must convert inert N_2_ into bioavailable ammonia. This additional metabolic burden can divert energy and resources away from growth and polymer synthesis, resulting in diminished PHB yields that may be impractical for large-scale industrial applications. Studies indicate that continuous cultivation under diazotrophic conditions can inhibit the metabolic activity of pure MOB strains, further curtailing their PHB synthesis capability. This suppression may be caused by alterations in central metabolic fluxes or the inhibition of key enzymatic activities under prolonged N_2_ fixation (Dubey et al., 2021). These findings highlight that the use of N_2_ as a sole nitrogen source involves significant trade-offs between environmental benefits and production efficiency.

The influence of N_2_ as a nitrogen source on the microbial synthesis of PHB from methane is multifaceted. Under specific conditions, N_2_ can enhance PHB accumulation by modulating key metabolic pathways, inducing essential enzyme synthesis, and promoting intracellular homeostasis (Müller-Santos et al., 2021). Conversely, its use presents significant challenges, such as reduced microbial growth rates and potential inhibition of metabolic activity. For practical implementation, a balanced strategy is required. This involves optimizing cultivation parameters and carefully regulating nitrogen supply to maximize PHB yield while mitigating the inherent limitations of N_2_ fixation (Bao et al., 2021). Developing such optimized processes is critical for advancing the industrial-scale production and sustainable application of PHB.

### 3.3 Metabolic pathways

The architecture and regulation of metabolic pathways fundamentally influence the efficiency of PHB biosynthesis from single-carbon and N_2_ sources. Key factors include the intracellular flux of carbon substrates and the precise control of metabolic nodes within the network, which are critical for directing carbon toward polymer accumulation (Behera et al., 2022). To enhance this bioconversion, metabolic engineering strategies are being employed to optimize these pathways, thereby facilitating the effective channeling of methane-derived carbon into PHB. This can be achieved by modulating the expression of genes encoding pivotal enzymes, such as those involved in acetyl-CoA formation and PHB polymerase, to boost catalytic activity and flux. A thorough investigation of the MOB metabolic network is therefore essential to fully elucidate the cellular mechanisms governing PHB synthesis, which will provide a foundation for scaling this process for industrial application (Wang et al., 2021).

**Figure 1:**
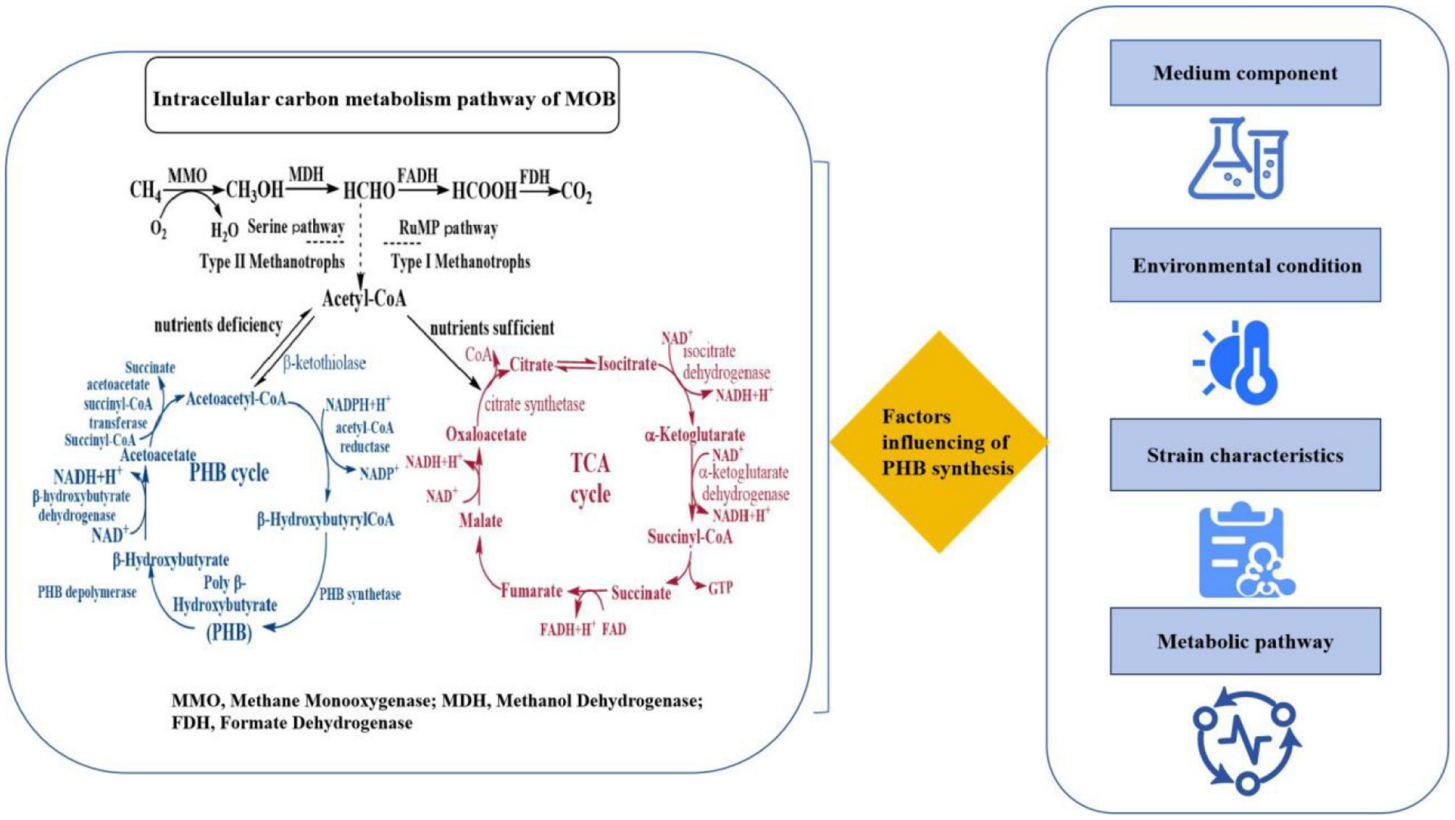
Factors influencing of PHB synthesis.

The metabolic pathway for PHB synthesis from acetyl-CoA in MOB is governed by three key enzymes, which are highly conserved across PHB-producing bacteria and represent critical nodes for metabolic engineering (Figure 1 and Table 2). The first committed step is catalyzed by β-ketothiolase (encoded by the *phbA* gene), which condenses two molecules of acetyl-CoA into acetoacetyl-CoA. This enzyme is often subject to feedback inhibition by high concentrations of CoA, linking its activity directly to the cellular energy status. The subsequent step is the stereospecific reduction of acetoacetyl-CoA to (R)-3-hydroxybutyryl-CoA, a reaction driven by NADPH-dependent acetoacetyl-CoA reductase (encoded by *phbB*). The availability of NADPH, a key reducing equivalent generated through methane oxidation and the pentose phosphate pathway, is therefore a major rate-limiting factor for PHB synthesis. The final and most critical enzyme is PHB synthase (encoded by *phbC*), which catalyzes the polymerization of (R)-3-hydroxybutyryl-CoA monomers into the high-molecular-weight PHB polymer. This membrane-associated enzyme is notoriously slow and is considered the primary bottleneck in the entire pathway; its activity and expression are strongly upregulated under conditions of nutrient imbalance, particularly nitrogen limitation.

**Table 2:** Key enzymes, genes, and regulatory factors in the PHB synthesis pathway of MOB.

**Metabolic pathway/step**	**Key enzyme**	**Encoding gene(s) (e.g., in *Methylococcus capsulatus* bath)**	**Function/reaction catalyzed**	**Known regulatory factors & notes**
**Methane oxidation**	Methane monooxygenase (pMMO)	*pmoCAB* operon	CH4 + O_2_ + H^+^ + e^−^ → CH3OH + H_2_O	Copper (Cu^2^^+^) availability; O_2_ concentration; N-source limitation can divert carbon flux.
**Methanol oxidation**	Methanol dehydrogenase (MDH)	*mxaFJGIR* (or *xoxF*)	CH3OH → HCHO (Formaldehyde)	Calcium (Ca^2^^+^) or Lanthanides (for xoxF); pivotal branch point in metabolism.
**Formaldehyde assimilation**				
Serine pathway	Hydroxypyruvate reductase	*hpr*	Glyoxylate + NADH → Glycine	Predominant in Type II methanotrophs.
Ribulose monophosphate (RuMP) pathway	Hexulose-6-phosphate synthase	*hps*	HCHO + Ru5P → Hu6P	Predominant in Type I methanotrophs
**Precursor formation**				
	Pyruvate dehydrogenase complex	*pdh*	Pyruvate → Acetyl-CoA + CO_2_	Key node supplying central precursor for PHB synthesis.
**PHB-specific synthesis**				
**Step 1: condensation**	β-Ketothiolase	*phbA (bktB)*	2 Acetyl-CoA → Acetoacetyl-CoA	Competitive pathways (TCA cycle); regulated by energy charge and NADH/NAD^+^ ratio.
**Step 2: reduction**	Acetoacetyl-CoA reductase	*phbB*	Acetoacetyl-CoA + NADPH → (R)-3-Hydroxybutyryl-CoA	NADPH/NADP^+^ ratio; carbon excess and nitrogen limitation strongly induce expression.
**Step 3: polymerization**	PHB synthase	*phbC*	(R)-3-Hydroxybutyryl-CoA → PHB + CoA	Substrate availability; post-translational regulation; critical for polymer chain length.
**N**_**2**_ **fixation**	Nitrogenase	*nifHDK*	N_2_ + 8H^+^ + 8e^−^+ 16 ATP → 2NH3 + H_2_ + 16ADP + 16Pi	Oxygen sensitivity (requires microaerobic conditions); Ammonia repression; Nickel (Ni) and Iron (Fe) cofactors.

The efficiency of PHB synthesis from one-carbon and N_2_ resources is contingent on a variety of factors. Optimizing these factors can enhance the efficiency of PHB synthesis from one carbon and N_2_ resources. With the continuous development of molecular biology and bioengineering technology, further in-depth exploration and solution of the above key issues will provide new possibilities for the industrial production of PHB (Fernandez-Bunster and Pavez, 2022), thus promoting the sustainable development and application of biomaterials.

### 3.4 Factors affecting the efficiency of PHB synthesis

A multitude of factors have been identified as contributors to the efficiency of PHB synthesis from one carbon and N_2_ resources. These include the composition of the medium, the environmental conditions, the characteristics of the strain, and the metabolic pathways involved (Figure 1).

#### 3.4.1 Media composition

The formulation of the culture medium is a critical factor governing the efficiency of PHB biosynthesis from one carbon and N_2_ sources. Synthesis efficiency is highly dependent on the specific types and concentrations of substrates supplied. For instance, the addition of complex nutrients such as yeast extract, glucose, or specific amino acids can stimulate microbial metabolism and promote PHB accumulation. Consequently, the systematic optimization of medium composition is an essential strategy for enhancing PHB yield and overall process efficiency.

#### 3.4.2 Growth conditions

Environmental conditions, including temperature, pH, and nutrient composition, can influence the efficiency of PHB synthesis from one carbon and N_2_ source. Temperature is a crucial factor that affects microbial growth and metabolic processes. Cantera et al. (2023) demonstrated that distinct MOB strains exhibit varying degrees of temperature adaptation. However, MOB is generally capable of enhanced growth rates and PHB synthesis within an optimal temperature range (typically 30 °C to 37 °C). Alterations in pH levels influence intracellular metabolic processes, and the susceptibility of different MOB to pH fluctuations varies. The accumulation of PHB is typically favored by maintaining neutral or slightly alkaline conditions. Furthermore, the management of dissolved oxygen is of paramount importance. A deficiency in dissolved oxygen can impede the oxidation efficiency of methane, consequently influencing the synthesis of PHB. It is therefore essential to establish appropriate culture conditions to maintain optimal environmental parameters, which facilitate the determination of the efficiency of PHB synthesis in MOB.

##### 3.4.2.1 Effect of temperature on PHB synthesis

The temperature is a significant factor influencing microbial growth and metabolic activities, which directly affects the efficiency of PHB synthesis from one carbon and N_2_ resource. It has been demonstrated that MOB demonstrate optimal growth rates and PHB synthesis capacity within a specific temperature range. Elevated or reduced temperatures typically result in a decline in the metabolic activity of the bacterium, which in turn affects the synthesis of PHB. In numerous experiments, the PHB synthesis of one carbon and N_2_ resources has exhibited the most favorable activity within the temperature range of 30 °C to 37 °C (Jo et al., 2021). In conditions conducive to optimal functioning, MOB can facilitate the provision of precursor materials through methane oxidation, thereby stimulating PHB synthesis. Furthermore, elevated temperatures augment the permeability of the cell membrane, which also facilitates substrate uptake. However, it should be noted that the optimal temperature range may not be maintained, which could potentially impact the fluidity of the cell membrane and consequently disrupt the intracellular environment. This, in turn, may lead to a reduction in the synthesizing ability of PHB.

##### 3.4.2.2 Effect of pH on PHB synthesis

The pH of the culture medium is a critical parameter governing microbial metabolic activity. For MOB, an optimal pH range of 6.5 to 7.5 supports peak intracellular enzyme activity. This optimal enzymatic function, coupled with efficient substrate conversion, promotes enhanced PHB accumulation. Deviations from this range are detrimental; acidic conditions can inhibit essential enzymes, while alkaline environments may lead to ammonia toxicity, both suppressing growth and metabolism. Consequently, MOB growth rates and PHB yields vary significantly with pH fluctuations. The observed sensitivity indicates that strategic pH control is a viable approach for optimizing PHB biosynthesis from single-carbon and N_2_ resources (Salem et al., 2021).

##### 3.4.2.3 Effect of nutrients on PHB synthesis

Nutrient availability is of important significance for the promotion of microbial growth and metabolism, with sufficient carbon, nitrogen, and other trace elements being essential for the synthesis of PHB from carbon and nitrogen sources. In the context of MOB, methane constitutes the one carbon source, with nitrogen sources typically derived from ammonia or nitrate. The importance of trace elements, including magnesium, manganese, and zinc, in enhancing enzyme activity and cellular metabolism has been well-documented. Concentrations of trace elements have been demonstrated to have a significant impact on the growth rate and PHB synthesis of MOB (Cardoso et al., 2022). Furthermore, the appropriate carbon to nitrogen ratio (C/N ratio) is also a crucial factor in promoting PHB synthesis, and the accumulation of PHB can be significantly enhanced by optimizing the C/N ratio.

The study of PHB synthesis from one carbon and N_2_ resource demonstrated the significant impact of temperature, pH and nutrients on microbial metabolism. To enhance PHB production, comprehensive and systematic investigations under defined conditions are essential. By modulating temperature and pH, an optimal growth milieu can be established for MOB, while a balanced nutrient ratio can markedly enhance the synthesis efficacy of PHB. Future investigations into the mechanism of PHB synthesis in MOB under diverse environmental conditions will not only facilitate a comprehensive understanding of its metabolic pathway but also offer a viable industrialization trajectory for environmental remediation and biomaterial production.

#### 3.4.3 Strain phenotypes

In addition, the characteristics of the strain itself play a critical role in the efficiency of PHB synthesis. Significant interspecies variations exist among MOB in their metabolic pathways, physiological traits, and inherent capacity for polymer accumulation. For instance, certain species, such as those within the genera *Methylocystis* and *Methylosinus*, demonstrate high PHB yields even at low methane concentrations, whereas other strains exhibit markedly lower productivity. Therefore, the selection and development of high-performance methane-oxidizing strains is a fundamental strategy for enhancing PHB biosynthesis (Gesicka et al., 2024). This can be achieved through targeted genetic modifications to optimize metabolic flux or via the screening of robust strains adapted to specific environmental conditions, both approaches serving to significantly increase production.

## 4 Optimization strategies for PHB synthesis

The biosynthesis of PHB is influenced by multiple critical factors, encompassing strain selection, culture condition optimization, and the provision of suitable single-carbon and nitrogen sources (McAdam et al., 2020). Key objectives for its industrial production include enhancing synthesis efficiency, reducing costs, and improving polymer quality. Consequently, research efforts have been directed toward developing interdisciplinary optimization strategies integrating microbiology, biochemistry, and biotechnology. These approaches aim to augment PHB yield and quality through process refinement, metabolic pathway modulation, and culture system design. A summary of PHB synthesis from MOB utilizing one carbon and N_2_ resources is presented in Table 3.

**Table 3:** Study on the synthesis of PHB by MOB based on carbon and nitrogen sources.

**Strain**	**Carbon sources**	**Fermentation mode**	**Feature**	**Maximum PHB production (g/L)**	**References**
*Methylosinus trichosporium* OB3b	Methane and CO_2_	Batch	Utilizes methane as primary carbon, can fix CO_2_, growth-coupled PHB synthesis	1.8	Takeguchi et al., 1998
*Methylococcus capsulatus* Bath	Methane and methanol	Fed-batch	Tolerates high methane concentrations, methanol addition enhances PHB production	3.2	Nguyen et al., 1994
*Methylomonas* sp. JLW1	Methane and formate	Continuous	Efficient conversion of methane and formate, stable PHB production over long-term operation.	2.4	Rostkowski et al., 2013
*Methylosarcina fibrata* CSC1	Methane and acetate	Batch	Acetate supplementation improves cell growth and PHB accumulation.	4.1	Eam et al., 2024
*Methylobacterium extorquens* AM1	Methanol and CO_2_	Fed-batch	Can utilize methanol and CO_2_, shows high PHB productivity under specific nutrient conditions.	5.6	Kim et al., 2010
*Methylomicrobium alcaliphilum* 20Z	Methane and nitrate	Batch	Utilizes nitrate as nitrogen source, PHB synthesis related to methane and nitrate utilization	1.2	De Bont and Mulder, 1974
*Methylophilus methylotrophus* AS1	Methanol and ammonia	Continuous	High cell density culture, methanol and ammonia regulation affects PHB production.	7.3	Taylor et al., 2018
*Methylosinus sporium* 5	Methane and glycine	Batch	Unusual carbon and nitrogen combination, shows unique PHB synthesis characteristics.	0.9	Markowska and Michalkiewicz, 2009
*Methylocystis parvus* OBBP	Methane and urea	Fed-batch	Urea as nitrogen source, PHB synthesis with methane utilization and urea metabolism.	2.7	Rostkowski et al., 2013
*Methylomonas denitrificans* FJG1	Methane and nitrite	Batch	Nitrite reduction and PHB synthesis in the presence of methane	1.5	Fitch et al., 1996
*Methylosinus taiwanensis* LCY	Methane and alanine	Batch	Specific PHB synthesis pattern with methane and alanine as substrates.	1.6	Miyaji, 2011

### 4.1 Genetic engineering and metabolic engineering

To advance the efficiency of PHB biosynthesis from one carbon and N_2_ resources, the development and implementation of targeted genetic and metabolic engineering strategies is crucial. These approaches are directed toward optimizing microbial production pathways.

#### 4.1.1 Application of gene-editing techniques

Gene editing technology refers to the targeted modification of DNA in organisms through specific methods. In recent years, CRISPR-Cas9 technology has been the preferred choice of the majority of researchers due to its simplicity (Wu et al., 2020), specificity and high efficiency. With the advent of CRISPR-Cas9, gene editing technology has become a widely used tool in the scientific community. The design of specific guide RNAs enables double-stranded breaks to be made at the position of target genes, which can then be repaired by the cell's own mechanisms, thus facilitating knock-in or knock-out of genes. The use of gene editing technology to genetically modify MOB can lead to a significant improvement in its ability to synthesize PHB. PHB synthesis is a complex process that involves the interaction of multiple metabolic pathways. Gene editing technology offers the potential to target specific genes and regulate the expression of key enzymes. The nature of key enzymes exerts a significant influence on the production of PHB, and consequently, the efficiency of PHB synthesis. The conversion of methane to PHB can be facilitated by reconfiguring its metabolic network utilizing synthetic biology approaches. This can be achieved in two ways: by enhancing the synthesis of precursors, and by inhibiting unwanted metabolic pathways, thus converting the carbon source to PHB in a more efficient manner.

The synthesis of PHB in MOB involves three key enzymes: β-ketothiolase (encoded by the *phbA* gene), which catalyzes the condensation of two molecules of acetyl-CoA to form acetoacetyl-CoA; acetoacetyl-CoA reductase (encoded by the *phbB* gene), which reduces acetoacetyl-CoA to D-3-hydroxybutyryl-CoA; and poly (hydroxybutyrate) synthase (encoded by the *phbC* gene), which is responsible for polymerizing D-3-hydroxybutyryl-CoA to form PHB. 3-hydroxybutyryl coenzyme A to form PHB (Anderson and Dawes, 1990). Researchers have overexpressed or optimized these key enzyme genes through a variety of genetic engineering tools. For example, in *Bacillus methanolicus*, the overexpression of the *phbA, phbB*, and *phbC* genes was achieved by replacing the natural promoters of these genes with strong promoters. The results showed that compared with the wild-type strain, the engineered strain increased the production of PHB by about 30%, reaching about 40% of the dry weight of the cells (Wang et al., 2023). In addition, rational design and modification of the protein structure of key enzymes to improve their catalytic activity and stability are also important strategies for optimizing endogenous synthetic pathways. In one study, the active center of polyhydroxybutyrate synthase was modified by targeted mutagenesis to enhance its affinity for the substrate, thereby increasing the synthesis rate and yield of PHB (Borrero-de Acuña and Poblete-Castro, 2023).

The efficacy of these genetic engineering strategies is powerfully illustrated by several recent case studies that target specific genes with measurable outcomes on PHB production. For instance, in *Methylocystis* sp. SB2, the overexpression of the entire native *phbCAB* operon under a strong promoter led to a 50% increase in PHB accumulation compared to the wild-type strain, even under non-nitrogen-limiting conditions, demonstrating the bypass of natural regulatory constraints (Guo et al., 2022). In another study with *Methylobacterium extorquens*, knockout of the *ackA* gene (encoding acetate kinase), which competes for the central precursor acetyl-CoA, successfully redirected carbon flux toward PHB synthesis, resulting in a 30% higher yield (Yoon et al., 2022). Conversely, heterologous expression of the *phaJ* gene from *Pseudomonas putida* (encoding (R)-specific enoyl-CoA hydratase) in *Methylosinus trichosporium* OB3b created a novel bypass pathway that converts β-oxidation intermediates directly to PHB precursors, broadening the substrate range and enhancing polymer synthesis from diverse carbon sources (Kulkarni et al., 2024). These targeted interventions—enhancing native pathways, eliminating competing reactions, and introducing novel metabolic routes—provide concrete evidence of how precision genetic engineering can directly and effectively optimize the PHB synthesis potential of MOB.

##### 4.1.1.1 Introduction of heterologous genes to broaden substrate utilization and metabolic pathways

In addition to optimizing the endogenous synthetic pathway, the introduction of heterologous genes has broadened the scope of substrate utilization and metabolic pathway of MOB, which opens up new avenues for increasing the production of PHB. Many MOB are naturally only capable of utilizing one-carbon compounds such as methanol, but the ability to synthesize PHB from multiple carbon sources can be made possible by introducing genes capable of utilizing other sugars through genetic engineering means. For example, introducing the genes encoding phosphoenolpyruvate carboxylase and transketolase from *E. coli* into methylotrophic bacteria constructed a new carbon metabolism pathway that enabled the engineered strains to utilize glucose as a carbon source for the synthesis of PHB (Ray et al., 2023). It was shown that the growth rate and PHB production of the engineered strains in the medium with glucose as the carbon source were significantly higher than those in the culture conditions with methanol as the carbon source only. In addition, heterologous genes encoding key enzymes of other metabolic pathways can be introduced to alter the distribution of metabolic flow in MOB and promote the flow of more carbon sources to the PHB synthesis pathway. The introduction of a pyruvate dehydrogenase bypass gene into MOB can increase the supply of acetyl coenzyme A, which provides more substrates for PHB synthesis, thus increasing PHB production (Yoon et al., 2022).

##### 4.1.1.2 Complex metabolic regulatory networks

The metabolic regulatory network of MOB is extremely complex, and many genes are involved and interrelated. Although some key genes and metabolic pathways have been investigated, the global regulatory mechanism of the whole metabolic network has not been fully analyzed. When genetically modified, a change in one gene may trigger a series of unexpected chain reactions that affect other physiological processes in the cell. For example, when overexpressing key genes of the PHB synthesis pathway, the original metabolic balance in the cell may be disrupted, leading to a decrease in cell growth rate. This is because PHB synthesis consumes a large amount of energy and carbon sources, and over-allocation of resources to PHB synthesis can interfere with the synthesis of other substances (e.g., proteins, nucleic acids, etc.) required for cell growth (Pham et al., 2022). In addition, there are multiple transcriptional regulators and signaling pathways in MOB that finely regulate gene expression and metabolic pathways. In the process of engineering modification, how to precisely regulate these regulatory factors and signaling pathways to achieve effective activation of the PHB synthesis pathway without affecting the normal physiological functions of the cells is an urgent challenge to be resolved.

##### 4.1.1.3 Environmental sensitivity

MOB is extremely sensitive to the environmental conditions of fermentation, and small changes in temperature, pH, substrate concentration, dissolved oxygen and other factors may significantly affect its growth and PHB synthesis ability. Too high or too low a temperature can affect the activity of MOB intracellular enzymes, which in turn affects the rate of cellular metabolism and the efficiency of PHB synthesis. For example, for some thermophilic MOB, when the fermentation temperature is higher than the optimal growth temperature (usually 30 −37 °C), the structure and function of intracellular proteins and cell membranes are impaired, resulting in stunted cell growth and decreased PHB production (Mokhtari-Hosseini et al., 2009). Therefore, how to precisely control the fermentation environment conditions and maintain the stable and efficient PHB synthesis capacity of MOB is a major challenge for industrial production.

##### 4.1.1.4 Genetic modification technical challenges

The gene integration methods, such as homologous recombination techniques, are often inefficient in MOB, making it difficult to obtain a large number of engineered strains that stably integrate exogenous genes. This may be due to the special cell wall structure of MOB, which impedes the uptake and integration of exogenous DNA, or the strong degradation of exogenous DNA by its own endonuclease system (Sauvageau et al., 2024). The genetic stability of the engineered strains is poor during continuous passaging culture, and the integrated exogenous genes may be lost or mutated, resulting in a gradual decrease in the PHB synthesis ability of the engineered strains. This may be related to the DNA repair mechanism in MOB cells as well as the instability of plasmid. In MOB, the precise regulation of gene expression is also a difficult issue, and the expression levels of different genes need to be precisely coordinated to achieve efficient PHB synthesis. However, the current commonly used gene expression regulatory elements (e.g., promoters, terminators, etc.) do not work well in MOB, and it is difficult to achieve precise control of gene expression, which affects the optimization of the performance of engineered strains.

Addressing these challenges requires the development of more sophisticated genetic tools tailored for MOB. Promising candidates for genetic modification extend beyond the core *phbCAB* genes to include global regulators (e.g., genes encoding the *Ntr* system) to decouple growth from PHB production, as well as genes involved in cofactor regeneration (e.g., to enhance NADPH supply for *PhbB*). In terms of methodology, while CRISPR-Cas systems are the primary focus, exploring alternative techniques such as recombinase-assisted genome engineering or transposon-based delivery might offer advantages for specific applications. Future efforts should prioritize creating a versatile and efficient genetic toolbox for MOB to unlock their full potential for metabolic engineering.

#### 4.1.2 MOB existing research on metabolic engineering

##### 4.1.2.1 Application of CRISPR, TALEN and ZFN technology in MOB gene editing

The CRISPR—Cas system, as an emerging gene editing technology, has been widely used in the field of MOB metabolic engineering in recent years. It utilizes a guide RNA complementary to the target gene to guide the Cas nuclease to recognize and cleave the target gene, realizing the knockout, insertion or substitution of the gene (Pham et al., 2022). In MOB, genes related to the PHB synthesis pathway and other metabolic regulatory genes can be efficiently edited by constructing suitable CRISPR-Cas9 or CRISPR-Cas12a systems. For example, knocking out the gene encoding a negative regulator in MOB using the CRISPR-Cas9 system lifted the inhibition of the PHB synthesis pathway, resulting in an increase of about 50% in PHB production in the engineered strain. Compared with traditional gene editing methods, CRISPR technology has the advantages of simple operation, high editing efficiency and high specificity, providing a more precise and efficient tool for metabolic engineering of MOB. However, in practical applications, CRISPR technology also faces some challenges, such as off-target effects that may lead to unintended gene editing and affect the normal physiological functions of the cells. Additionally, how to introduce the CRISPR system into MOB cells efficiently and achieve stable gene editing requires further optimization of the experimental conditions and technical means.

In contrast, the application of earlier engineered nuclease systems, such as Zinc-Finger Nucleases (ZFNs) and Transcription Activator-Like Effector Nucleases (TALENs), in MOB—and particularly in model strains like *Methylococcus capsulatus* Bath—is exceedingly limited and rarely reported. The primary hurdle for all these technologies, including CRISPR-Cas, is the development of a efficient and reliable genetic toolbox. The range of available shuttle vectors for *M. capsulatus* is narrow, and standard transformation methodologies like electroporation often suffer from low efficiency (Kotnik et al., 2015). Conjugation from *E. coli* is a more common but labor-intensive alternative. A critical barrier is the host's potent restriction-modification (R-M) systems, which can degrade incoming plasmid DNA carrying the editing machinery. While CRISPR-Cas systems can be delivered as pre-assembled ribonucleoproteins (RNPs) to bypass some of these issues, the complex protein engineering required for ZFNs and TALENs makes their delivery even more challenging. Consequently, the practical application of ZFNs and TALENs in MOB has been hindered by these technical obstacles, with CRISPR-Cas emerging as the more feasible and actively pursued option for genetic manipulation in these bacteria.

##### 4.1.2.2 Pathway optimization based on metabolic modeling

In order to more comprehensively understand the metabolic network of MOB and the interrelationships among the metabolic pathways, the researchers constructed a variety of global metabolic models of MOB. These models integrate the genomic information, metabolic reactions and related physiological parameters of MOB, and predict the metabolic behaviors of cells under different conditions by mathematical simulation (Wutkowska et al., 2024). Based on metabolic modeling, researchers can analyze the contribution of different metabolic pathways to PHB synthesis and predict the effects of genetic modification and altered environmental conditions on the metabolic network to design targeted metabolic engineering strategies. For example, metabolic modeling analysis revealed that enhancing the flux of the pentose phosphate pathway in MOB could provide more reducing power (NADPH) and precursors for PHB synthesis, so the PHB production of the engineered strain was successfully increased by overexpression of key enzyme genes in the PPP pathway through genetic engineering.

In practical applications, the resources of carbon monoxide and nitrogen may be subject to stress due to changes in pH, salinity and temperature. The application of gene editing has the potential to enhance the environmental adaptability of microbial strains, thus facilitating their utilization in industrial production processes. Metabolic processes in microbial organisms are inherently complex, and the global metabolic effects of gene editing are often difficult to predict. Changes in environmental conditions can have a significant impact on the performance of transgenic strains. Consequently, a comprehensive investigation into the stability and adaptability of these strains is imperative for their potential utilization in industrial environments. From a safety perspective, the application of gene editing techniques must comply with a rigorous regulatory framework to ensure their safety in the wider environment and ecosystems (Piergentili et al., 2021). It is the responsibility of scientists to conduct a thorough risk assessment of gene editing and to conduct research within reasonable limits.

#### 4.1.3 The case for metabolic pathway optimization

The optimization of metabolic pathways in the production of PHB from one carbon and N_2_ resource has the potential to significantly enhance the production and conversion efficiency of PHB (Zhang et al., 2022). The purpose of pathway optimization is twofold: to improve the efficiency of biosynthesis and to ensure the long-term stable survival and development of microorganisms in the environment. In a study of a specific MOB (Hassan and Saleh, 2023), Hassan used genetic engineering to optimize its pathway, effectively reducing the energy loss of the cell during the synthesis of PHB. This was achieved by knocking out key genes that produce by-products unrelated to PHB synthesis. By increasing the expression of key enzymes, in particular hydroxyacetyl-CoA decarboxylase and D-3-hydroxybutyrate dehydrogenase, the efficiency of PHB synthesis was greatly improved.

It is evident from the investigation of the optimization of metabolic pathways for the synthesis of PHB from one carbon and N_2_ resource that MOB play a important role in maintaining ecological balance and demonstrate considerable potential for application in sustainable development.

### 4.2 Optimization of culture conditions

In the process of optimizing culture conditions, it is imperative to regulate various factors, including temperature, pH, and nutrients (Kopperi et al., 2021). Altering the medium composition has been shown to promote the proliferation of MOB and the accumulation of PHB. When these factors are given full consideration, the process of PHB synthesis from one carbon and nitrogen can be optimized, thus laying a solid foundation for its application in the development of renewable resources and the protection of the environment.

**Figure 2:**
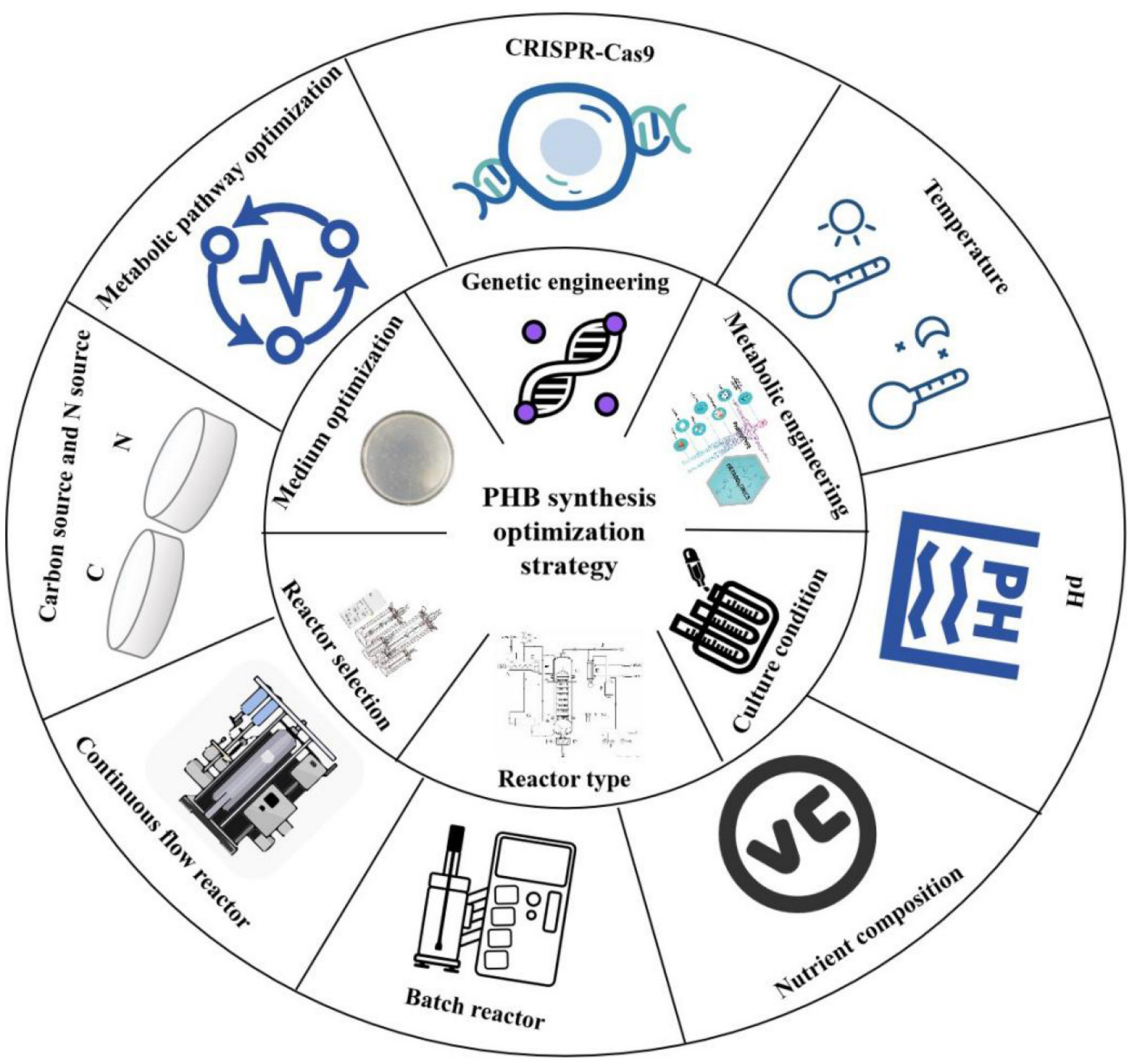
PHB synthesis optimization strategy.

Despite the considerable potential of various optimization strategies for enhancing PHB production in MOB, each approach presents distinct limitations and challenges that must be acknowledged. Genetic engineering strategies, while powerful, often encounter issues such as metabolic burden from gene overexpression, unintended pleiotropic effects from pathway modifications, and generally underdeveloped genetic tools for non-model methanotrophs, compounded by regulatory constraints surrounding GMO applications. Culture condition optimization, particularly nutrient limitation strategies, frequently improves PHB accumulation at the expense of biomass yield and overall volumetric productivity (Figure 2), with additional challenges in scaling up laboratory-optimized conditions to industrial bioreactors due to issues with nutrient and gas transfer homogeneity (Valentin and Steinbüchel, 1994). Reactor design and process configuration face inherent trade-offs between productivity, operational stability, and energy consumption, especially concerning gas mass transfer limitations inherent to methane-based systems. These collective challenges underscore the necessity of an integrated approach that combines robust strain development with economically viable bioprocess design to advance toward commercial implementation.

### 4.3 Reactor types and their selection

The principal reactor types employed in the manufacture of PHB encompass batch reactors, continuous flow reactors, sequential batch reactors, and batch flow reactors (Lai et al., 2022). The batch reactor represents the simplest reactor type, exhibiting a relatively straightforward operational process, which renders it well-suited to small-scale production. This reactor performs the reaction for a fixed time following the simultaneous injection of all substrates, after which product collection and reactor cleaning are conducted at the conclusion of the reaction. The batch reactor is an effective tool for reducing the risk of foreign contamination in the production of PHB from one carbon and N_2_ sources. Batch reactors are characterized by their operational simplicity, allowing for precise control of variables, which facilitates fundamental research and process optimization. However, their productivity is constrained by the inherent downtime between cycles for loading and unloading. In contrast, continuous-flow reactors operate with a constant inflow of substrates and outflow of products. This configuration maintains a stable metabolic environment suitable for prolonged operation, enabling high cell densities and significantly enhanced production efficiency. The constant methane supply supports consistent PHB synthesis, while integrated real-time monitoring systems make these reactors ideal for large-scale industrial applications (Jayakrishnan et al., 2021). Sequencing batch reactors incorporate features of both batch and continuous systems by executing discrete processing steps within a single vessel during each cycle, thereby improving operational flexibility and efficiency. A potential disadvantage is the increased complexity of control and management. Conversely, conventional batch reactors offer superior flexibility for small-scale laboratory studies. This format allows for direct and facile adjustment of media composition and environmental parameters to accommodate the specific metabolic demands of diverse microbial strains.

The selection of a bioreactor for PHB production involves critical trade-offs among scalability, economic viability, and environmental footprint. Batch reactors offer operational simplicity and low contamination risk, yet their scalability is limited. Significant unproductive downtime results in low volumetric productivity, rendering them cost-ineffective and energy-intensive for large-scale applications. Fed-batch operations improve upon this by enabling higher cell densities and superior substrate utilization, but they face comparable scalability constraints and increased operational complexity. In contrast, continuous stirred-tank reactors (CSTRs) demonstrate excellent scalability and higher productivity. Their steady-state operation enhances cost-effectiveness and resource utilization. Nevertheless, CSTRs necessitate considerable capital investment, advanced control systems, and are susceptible to contamination and genetic instability. Bubble column and airlift reactors provide a more energy-efficient alternative, utilizing passive mixing to lower operational costs and environmental impact. However, their scalability can be hindered by mass transfer limitations and the development of concentration gradients. The optimal reactor choice thus necessitates balancing these competing factors. CSTRs are often preferred for large-volume production due to their high productivity, despite higher initial costs. Conversely, bubble column reactors present a more sustainable alternative for processes where mass transfer rates are adequate.

The selection of an appropriate bioreactor is governed by multiple engineering and biological parameters. Key considerations include reactor volume, gas-liquid mass transfer efficiency, mixing intensity, and temperature control. Furthermore, the construction material and internal geometry significantly influence MOB proliferation and subsequent PHB productivity. Systematic experimentation and optimization during the design phase are therefore essential to establish an efficient production system. Since MOB requires specific environmental conditions for optimal growth and metabolism, the ultimate reactor choice must be predicated on their inherent biological characteristics. The primary objective is to create a controlled environment that supports robust microbial culture and directs metabolic activity toward maximizing PHB yield.

#### 4.3.1 Key factors in reactor selection

For small-scale experimental studies, batch reactors are typically employed, as they facilitate parameter optimization through precise control of environmental conditions. In contrast, large-scale industrial production often utilizes continuous-flow reactors to maximize volumetric productivity. The decision must account for the specific culture requirements, metabolic characteristics, and growth kinetics of the MOB. Continuous-flow reactors are particularly advantageous for processes prioritizing high methane conversion efficiency, as they maintain steady-state conditions. Alternatively, sequential batch reactors offer superior control over substrate feeding and reaction phases, making them suitable for processes requiring precise metabolic regulation.

##### 4.3.1.1 Economy

A comparison of the economic benefits of different reactors will facilitate the identification of the optimal solution, thereby enabling the sustainable development of MOB synthesis for PHB production. Among the various reactor types, the continuous flow reactor exhibits superior efficiency; however, it is associated with relatively high initial investment and maintenance costs (Su et al., 2022). In addition, the scale-up design of the reactor should also take into account the accessibility of feedstock, the treatment of waste products and the potential impact of the technology on the environment. For instance, the selection of a continuous flow reactor or a batch reactor will have a significant effect on the utilization of feedstock and the consumption of energy. It is only through a comprehensive economic analysis that the commercial viability and environmental friendliness of the technology can be guaranteed. In practice, the selection of an appropriate reactor necessitates not only an evaluation of the initial investment and operational costs, but also an assessment of the long-term viability and environmental impact of the chosen technology. From one perspective, a sustainable production process will prevent the secondary pollution of the environment. From another perspective, it provides an advantage for companies to compete in the market. Furthermore, the reactor footprints, ease of operation and degree of automation also have an impact on the overall economics to a certain extent, as these factors can influence labor costs and operational efficiency. When these factors are taken into consideration, a reasonable reactor selection will help to optimize the economic benefits of synthesizing PHB from one carbon and N_2_ resources, thus promoting its application and development in industrial production.

##### 4.3.1.2 Self-conditioning of the reactor

The material and design of the reactor are equally important for the efficiency of PHB synthesis using one carbon and N_2_ resources. When selecting materials, corrosion resistance and ease of cleaning should be prioritized in order to prevent the introduction of exogenous contamination during the reaction process. The flow characteristics of gases and liquids should also be considered to ensure optimal reaction performance. The hydrodynamic conditions within a bioreactor, governed by its configuration, including geometry, scale, and port placement, directly influence mass transfer efficiency. Effective mixing is critical, as it ensures homogeneous distribution of gaseous substrates like methane and oxygen. This homogeneity enhances the rate of methane uptake and consequently accelerates cellular metabolism, which is directly linked to the growth of MOB and the synthesis of PHB.

Operational parameters exert significant influence on PHB biosynthesis. Temperature directly impacts both the growth rate of MOB and the enzymatic efficiency of PHB synthesis; an optimal temperature enhances cellular metabolism, thereby promoting polymer accumulation. Similarly, pH variation can alter key metabolic pathways, and maintaining a stable pH is crucial for consistent cell growth and product formation. Furthermore, the gas flow rate must be carefully regulated to ensure effective mass transfer and homogeneous distribution of substrates within the reactor. Consequently, precise control of temperature, pH, and gas flow through integrated monitoring systems is necessary to maintain optimal conditions for PHB production.

The synthesis of PHB from one carbon and nitrogen resources represents a promising sustainable bioprocess. Within this framework, the selection of an appropriate bioreactor is a critical determinant of bioprocess efficiency and final polymer characteristics (McAdam et al., 2020). Strategic reactor design is essential for achieving high-yield, high-quality PHB production from MOB. Continued advancement, driven by progress in biotechnology and bioreactor engineering, is expected to yield further innovations. These developments will enhance production metrics and solidify the role of this technology in achieving circular economy objectives (González-Rojo et al., 2024).

### 4.4 Optimization of the culture medium

#### 4.4.1 Selection and optimization of carbon sources

Methane functions as the principal carbon substrate in bioprocesses utilizing one carbon and N_2_ resources. Determining the optimal methane concentration is essential and depends on the specific physiological traits of MOB and the prevailing culture conditions. While a range of 10% to 50% (v/v) is commonly employed, significant variation exists among strains in their methane tolerance and assimilation efficiency. For example, strains achieve peak growth and PHB production at 20% methane, whereas others are adapted to thrive at higher concentrations (Fergala et al., 2018). To augment microbial performance, supplementary one carbon compounds like methanol or formic acid can be introduced. The moderate addition of methanol, in particular, has been observed to enhance both biomass yield and intracellular PHB content. This is attributed to methanol's role as a readily assimilated substrate that can support growth and be directly channeled into the PHB biosynthesis pathway (Yoon et al., 2022). A critical consideration in mixed-carbon cultivation is the precise calibration of the carbon source ratio. This is necessary due to the distinct metabolic pathways and rates associated with different substrates, which can significantly influence overall cellular metabolism and product yield.

#### 4.4.2 Optimization of nitrogen sources

N_2_ serves as the primary nitrogen source, while small quantities of supplemental nitrogen, such as ammonium or nitrate, can be introduced to support distinct growth phases. This strategy ensures adequate nitrogen availability, enhancing both the growth rate and PHB synthesis capacity of the strain. However, nitrogen concentration must be carefully regulated to prevent the inhibition of key metabolic pathways. The choice of nitrogen source significantly influences MOB physiology; for instance, potassium nitrate has been shown to be superior to ammonium chloride in promoting growth and PHB accumulation in certain strains. The metabolism and PHB synthesis in MOB can be affected by an excess or paucity of nitrogen source. It is often necessary to determine the appropriate concentration range of the nitrogen source, typically between 0.5 and 5 g/L, through experimental means. In nutrient-balanced-nutrient-limited two-stage cultures, the optimization of the concentration of the nitrogen source can increase the amount of PHB without inhibiting cell growth. It has been demonstrated that when N_2_ was used as the nitrogen source, the PHB synthesis capacity of MOB was comparable at different methane-oxygen ratios. Screening and subsequent enrichment of efficient MOB was performed under conditions using N_2_ as a nitrogen source with a 2:3 methane to oxygen ratio. It was also determined that other dominant heterotrophic bacteria in the mixed colony may be able to utilize intermediates of methane oxidation to promote growth and PHB synthesis. In addition, the nitrogen concentration in the medium can be more precisely controlled using batch replenishment of nitrogen sources or continuous flow addition of nitrogen sources. It is recommended that an adequate nitrogen source be provided during the early stages of cell growth to support rapid cell expansion, followed by a controlled reduction in the supply of nitrogen as the cells enter the stabilization phase, thereby encouraging the cells to utilize a greater proportion of the carbon source for PHB synthesis (Garcia-Gonzalez et al., 2015). Similarly, amino acids such as glutamic acid and aspartic acid have been shown to function as growth factors and promote MOB growth. In conclusion, N_2_ represents an optimal nitrogen source for PHB synthesis by MOB.

#### 4.4.3 Adjustment of inorganic salts and trace elements

Macronutrients, including potassium, magnesium, calcium, and phosphorus, are fundamental medium components. They supply essential elements for cellular biosynthesis and help maintain optimal osmotic pressure and pH. Concurrently, trace elements such as iron, copper, zinc, and manganese serve critical roles as enzyme cofactors, significantly influencing methanotrophic activity. A key example is copper, which is a central component of pMMO. Its presence directly enhances the enzyme's catalytic activity, thereby increasing the efficiency of methane oxidation and subsequent utilization.

#### 4.4.4 Addition of growth factors

Research indicates that specific vitamins, such as vitamin B_12_ and biotin, function as essential micronutrients for the optimal metabolic function of MOB (Palacios et al., 2014). Empirical evidence confirms that supplementing methane-based media with vitamin B_12_ can markedly enhance both the growth rate and PHB yield in these microbial cultures.

#### 4.4.5 Adjustment of pH value

The initial pH of the culture medium is a critical determinant of growth and metabolic activity in MOB. An optimal pH range for most strains falls between 6.5 and 8.0. Specific strains, such as those within the genus *Methylobacterium*, demonstrate peak PHB accumulation and cellular growth at a more narrow initial pH of 7.0 to 7.5 (Kaparullina et al., 2025). Microbial metabolism during incubation often causes the medium's pH to fluctuate. To counteract this, buffers like dipotassium hydrogen phosphate and sodium carbonate can be incorporated. These compounds maintain pH stability, ensuring consistent growth and reliable PHB synthesis within a controlled acid-base environment.

#### 4.4.6 Optimizing the training model

The addition of carbon and other nutrients in a batch replenishment process circumvents the inhibitory impact of elevated nutrient concentrations on cellular growth, while simultaneously ensuring the provision of sufficient nutrients to enhance cell density and PHB production. For instance, during fermentation, methane and other nutrients are introduced in regular batches, contingent upon the stage of cell growth and nutrient consumption. Moreover, nutrient-balanced, nutrient-limited two-stage cultures have been demonstrated to favor PHB synthesis. In the nutrient-balanced phase (Gahlawat et al., 2020), an adequate supply of nutrients is provided to facilitate rapid cell growth. Conversely, in the nutrient-limited phase, the availability of nitrogen and other nutrients is restricted, prompting the cells to divert a greater proportion of carbon sources toward the synthesis of PHB for storage (Figure 2).

## 5 Future prospects

### 5.1 Potential for PHB synthesis in MOB with one carbon and N_2_ resources

It is anticipated that in the future, more in-depth modification of MOB through metabolic engineering will emerge as a key trend. Scholars will conduct further analysis of the metabolic pathway of PHB synthesis by MOB utilizing nitrogen, with the aim of clarifying the key enzymes and regulatory nodes. The precise regulation of these key genes is expected to be achieved through the use of gene editing technology, such as the CRISPR-Cas system. This is anticipated to enhance the assimilation efficiency of nitrogen by the strain and increase the synthesis flux of PHB. For instance, the enhancement of gene expression associated with nitrogen fixation enables MOB to more effectively transform nitrogen into a readily accessible nitrogen source, thereby ensuring an adequate supply of nitrogen for PHB synthesis and consequently elevating PHB production (Zhou et al., 2022). A deeper understanding of the physiology and metabolic requirements of MOB will elevate the importance of precise cultivation control. Future optimization will extend beyond conventional parameters like temperature, pH, and dissolved oxygen. It will involve more nuanced strategies for nitrogen supply, including its mode of addition, concentration, and ratio relative to methane. The development of intelligent bioreactor systems is a critical next step. Such systems would enable real-time monitoring and adjustment of conditions to maintain an optimal environment for both MOB growth and PHB synthesis. Furthermore, comparative studies on different cultivation modes, such as continuous culture versus fed-batch culture are necessary to determine the most effective strategy for PHB production using N_2_. Identifying the optimal cultivation strategy is essential for enhancing overall process efficiency.

The biosynthesis of PHB using N_2_ as nitrogen source offers considerable promise for enhancing environmental sustainability. This approach facilitates the large-scale conversion of methane, a potent greenhouse gas, into biodegradable polymers, thereby directly mitigating methane emissions and contributing to climate change mitigation (Morais et al., 2022). A key environmental advantage of PHB is its biodegradability, which stands in stark contrast to the persistence of conventional plastics. It can be completely broken down in natural environments, preventing long-term pollution. Furthermore, in agricultural applications, PHB can be utilized in the production of biodegradable mulch films or as a matrix for slow-release fertilizers, where its controlled degradation rate helps improve nutrient management. This approach can enhance the utilization efficiency of fertilizers and mitigate the contamination of agricultural surface sources. In the field of biomedicine, the functional attributes of PHB can be further enhanced to align with the requirements of tissue engineering and controlled drug release. This could facilitate the development of novel biomedical materials (Fernandez-Bunster and Pavez, 2022). Moreover, in the domains of packaging materials and electronic materials, we will persist in broadening the scope of applications for PHB, facilitate the environmentally conscious advancement of associated industries, and make more significant contributions to the realization of the objective of sustainable development.

### 5.2 Directions and challenges for further research

It is recommended that further research be conducted to identify MOB that exhibit enhanced nitrogen utilization efficiency and PHB synthesis capabilities. In addition to examining samples from typical environments, it would be beneficial to investigate extreme habitats, including the deep sea, polar regions, and high-temperature hot springs. Investigating unique ecosystems can reveal microorganisms with novel metabolic pathways and adaptive traits, facilitating the discovery of high-performance strains. In parallel, researchers are utilizing targeted gene editing techniques, such as CRISPR-Cas9, to genetically engineer existing methanotrophic bacteria. This approach aims to enhance the expression of genes governing nitrogen assimilation and PHB synthesis, thereby improving overall strain efficacy (Wang et al., 2023). A deeper analysis of the metabolic regulatory network activated during N_2_ fixation growth is also essential. This requires elucidating the interactions between key metabolic nodes. Subsequent pathway optimization through metabolic engineering. By modulating key enzyme activities or redirecting metabolic flux can then increase the efficiency of carbon and energy channeling toward PHB synthesis. Furthermore, the introduction of novel metabolic pathways or gene clusters to expand the substrate utilization scope by the strain and improve PHB synthesis efficiency represents a promising avenue for investigation.

## 6 Conclusion

Significant advancements have been made in the field of synthesizing PHB, utilizing one carbon and N_2_ resources. The metabolic pathway of MOB and the mechanism of PHB synthesis have been better elucidated, and the important roles of one carbon metabolism and nitrogen fixation in these processes have been elucidated. PHB, as a biodegradable plastic, among other applications, presents a novel option for sustainable production. However, the slow growth of MOB leads to low production efficiency, the nitrogen fixation efficiency and the regulatory mechanism of nitrogen metabolism are not fully clarified, and the PHB extraction and purification methods need to be improved. Future breakthroughs in this field depend on interdisciplinary cooperation, focusing on core challenges such as strengthening the metabolic coupling efficiency of nitrogen fixation and PHB synthesis, and optimizing large-scale production processes through systems biology and artificial intelligence strategies. At the same time, exploring the combination of its environmental and economic benefits and promoting the closed-loop circular economy model from laboratory to practical application will be the key opportunity to realize its industrialization potential.
